# ID 43 - Aplicando a Pedagogia da Cooperação no Processo de Acolhimento e Adaptação Curricular em Mestrado Profissional em ATS

**DOI:** 10.5327/2237-9622.2025.v34s1.43

**Published:** 2025-11-25

**Authors:** Flavia Tavares Silva Elias, Luiza Siqueira do Prado, Fábio Otuzi Brotto

## Abstract

**Introdução:**

A Rede Brasileira de Avaliação de Tecnologias em Saúde (Rebrats) é uma das estratégias institucionais da Política Nacional de Gestão de Tecnologias em Saúde (PNGTS) para a implementação do campo da avaliação de tecnologias em saúde (ATS) no Brasil. A Rede integra mais de 100 Núcleos de Avaliação de Tecnologias em Saúde (Nats), a maioria nas Regiões Sul e Sudeste. Em 2022, o Ministério da Saúde (MS) lançou chamada para mestrados profissionalizantes na área de ATS e a proposta do Nats do Programa de Evidências para Políticas e Tecnologias em Saúde (Nats-Pepts) da Fiocruz Brasília foi selecionada.

O processo seletivo durou três meses e incluiu apenas profissionais das Regiões Centro-Oeste, Norte e Nordeste. Foram recebidas 170 inscrições, 89 validadas após análise dos documentos enviados. Foram selecionados 20 candidatos, 15 (75%) mulheres e 5 (25%) homens; 12 (60%) atuam na Região Centro-Oeste, 6 (30%) na Nordeste e 2 (10%) na Norte. Além disso, 12 (60%) participam de Nats ou Natjus. Os pré-projetos envolviam temas como vacinação em áreas remotas, avaliações pós-incorporação e de impacto orçamentário.

Considerando a diversidade das áreas de atuação dos estudantes, optou-se por trabalhar o acolhimento e a integração usando a Pedagogia da Cooperação, criada por Fábio Otuzi Brotto e coautorias. O objetivo dessa metodologia é favorecer o desenvolvimento de relacionamentos colaborativos em grupos, potencializando a inteligência coletiva e a autonomia pessoal.

A experiência deu-se em dois momentos do primeiro encontro da turma. No primeiro, trabalharam-se desafios da ATS nos tópicos: 1) Contexto econômico, social e cultural, 2) Uso dos métodos de ATS no nível regional das secretarias de saúde e dos Nats, 3) Aplicabilidade da ATS em processos decisórios no nível regional das secretarias de saúde e dos NatsS, e 4) Tradução/interação de conhecimentos na implantação dos processos de ATS. O diálogo em grupo possibilitou troca de experiências entre estudantes, que compartilharam situações comuns em suas áreas de atuação. Já no segundo, quatro práticas da Pedagogia da Cooperação foram trabalhadas: 1) Fazer Com-Tato, 2) Estabelecer Com-Trato, 3) Compartilhar In-Quieta-Ações, e 4) Fortalecer Alianças e Parcerias. O mediador especialista atuou com as dinâmicas da Aprendizagem Colaborativa, Diálogo, Comunicação Não Violenta, Jogos Cooperativos, entre outras. Os resultados foram: Com-Trato de convivência, criado a partir das expectativas dos estudantes; elenco de "perguntas quentes" (inquietações e dúvidas mais relevantes) para orientar a aplicação de conteúdos importantes para a turma, associados ao currículo da formação; e, por fim, os Mínimos Passos Elegantes, observados por cada um para que o cuidar prevaleça, criando-se ambiente afetivo e de promoção de felicidade no aprendizado mútuo.

Este foi um trabalho de adaptação curricular baseado na expectativa dos estudantes e na cooperação entre eles e com a coordenação do curso, valorizando os saberes de cada pessoa. No contexto do SUS, o uso desse tipo de abordagem pode ser muito benéfico, pois profissionais de diferentes níveis interagem para construir ATS enraizada nas diversas práticas de saúde. Além disso, há um esforço de decolonização da ATS pensando nas práticas dos territórios das áreas de atuação dos estudantes.

**Método:**

1) Proposta de formação, 2) Perfil da turma, 3) Propósito da iniciativa, 4) Atividades da Pedagogia da Cooperação, 5) Resultados do currículo cocriativo do curso. Equipamentos necessários: monitor para apresentação de eslaides e fotos.

